# Criteria for identification of advanced Parkinson’s disease: the results of the Italian subgroup of OBSERVE-PD observational study

**DOI:** 10.1186/s12883-022-02554-z

**Published:** 2022-01-28

**Authors:** Alessandro Stefani, Alessandro Tessitore, Nicola Tambasco, Giovanni Cossu, Maria Gabriella Ceravolo, Giovanni Defazio, Francesca Morgante, Silvia Ramat, Gabriella Melzi, Giuliana Gualberti, Rocco Merolla, Koray Onuk, Leonardo Lopiano

**Affiliations:** 1 Parkinson Center, Department System Medicine, Policlinico Tor Vergata, Univ Tor Vergata, Rome, Italy; 2 Department of Advanced Medical and Surgery Sciences, University of Campania “Luigi Vanvitelli”, Napoli, Italy; 3 Movement Disorders Center, Neurology Department, Perugia General Hospital and University of Perugia, Perugia, Italy; 4 Department of Neuroscience, SSD Neurophysiology and Movement Disorders, AOB Brotzu Cagliari, Cagliari, Italy; 5 Department of Experimental and Clinical Medicine, School of Medicine, Marche Polytechnic University, Ancona, Italy; 6 Department of Medical Sciences and Public Health, University of Cagliari and Neurology Unit, AOU Cagliari, Cagliari, Italy; 7 Neurosciences Research Centre, Molecular and Clinical Sciences Research Institute, St George’s University of London United Kingdom, London, UK; 8 Department of Clinical and Experimental Medicine, University of Messina, Messina, Italy; 9 Parkinson Unit, Azienda Ospedaliero-Universitaria Careggi, Florence, Italy; 10 AbbVie Srl, SR 148 Pontina, 04011 Campoverde, LT Italy; 11 AbbVie Inc., North Chicago, IL USA; 12 Department of Neuroscience “Rita Levi-Montalcini”, University of Torino, AOU Città Della Salute e Della Scienza - Torino, Torino, Italy

**Keywords:** Advanced Parkinson’s disease, Device-aided treatment, Quality of life

## Abstract

**Background:**

Frequency of Advanced Parkinson’s Disease (APD) and its clinical characteristics are still not well defined. Here, we aimed to assess APD prevalence in the Italian OBSERVE-PD cohort, as well as treatment eligibility to device-aided therapies (DAT), and to compare the APD clinical judgment with the established Delphi criteria.

**Methods:**

This sub-group analysis of the OBSERVE-PD study was performed on patients enrolled by 9 Movement Disorders centers in Italy. Motor and non-motor symptoms, PD characteristics, activities of daily living, and quality of life were assessed. Patient eligibility for DAT, response to current PD treatments, referral process, and the concordance between APD physician’s judgment and Delphi criteria were also assessed.

**Results:**

According to physician’s judgment, 60 out of 140 patients (43%) had APD. The correlation between physician’s judgment and the overall APD Delphi criteria was substantial (K = 0.743; 95%CI 0.633–0.853), mainly driven by a discrete concordance found for the presence of ≥ 2 h of daily OFF time, presence of troublesome dyskinesia, ≥ 5 times daily oral levodopa dosing, and activities of daily living limitation. Forty-four (73%) APD patients were considered eligible to DAT but only 18 of them (41%) used these therapies, while most patients, independently from their eligibility, continued to use 3–5 oral daily medications, due to fear of invasive solutions and need to have a longer time to decide.

**Conclusion:**

APD was frequent in the Italian OBSERVE-PD population. DAT in the eligible APD population proved to be underused, in spite of unsatisfactory symptoms control with oral medications in 67% of patients.

## Background

Parkinson’s disease (PD) is a progressive neurodegenerative disorder characterized by a heterogeneous spectrum of motor and non-motor symptoms leading to disability and poor quality of life despite best medical treatment [1].

Many attempts have been done to define the progression of PD based on the occurrence of motor complications or levodopa-resistant non-motor symptoms such as cognitive impairment and axial disturbances, namely gait, speech and postural disturbances [2]. With the development of device-aided therapies (DAT), the advanced Parkinson’s Disease (APD) stage refers to that time over disease course when motor and non-motor fluctuations and dyskinesia [2] impact on patient’s quality of life (QoL) [3] and caregiver burden [4].

APD definition is still controversial, and it is still largely defined on the basis of consensus opinion and thus with several caveats [5]. APD is widely accepted as a term to describe patients with motor complications, but many patients continue to progress in their disease to a later stage that it is not yet conventionally accepted [2]. Yet, it is also to be noted that the identification and standardization of APD criteria in MD clinics is not homogeneous among the MD Centers.

The Delphi criteria have been recently developed to assist the recognition of APD based on the presence of motor and non-motor fluctuations as well as on functional criteria. Nevertheless, the early identification of the advanced stage still remains an unmet need, especially considering the importance of the holistic management of PD based on motor, non-motor symptoms and functional disability control [5].

The Delphi criteria have been developed through a consensus built among movement disorders specialists regarding key patient characteristics suggesting the transition to APD and guiding the appropriate use of device-aided therapies in the advanced stage [6]. In clinical practice the Hoehn & Yahr (H&Y) scale is used to classify the PD stage; this scale is mainly focused on postural instability, but it neither captures motor fluctuations nor NMS, two important disease progression milestones. The Unified Parkinson’s Disease Rating Scale (UPDRS), developed to determine disease severity levels requires high clinical expertise and is less used among the general neurologists compared to Movement Disorders (MD) specialists [6].

The difficulty in the early and correct identification of the advanced stage could represent a confounding element in the assessment of the real prevalence of APD worldwide. Previous epidemiological studies demonstrated that approximately 10% of all PD patients have APD [7, 8]. According to a Spanish survey, 24.7% of 81 PD patients analyzed were in H&Y stage IV or V [9]. More recently, a German survey stated that about 20% of PD German patients are in an advanced stage [10].

To date, there is a limited knowledge about the frequency of APD in Italy and a low awareness of the importance to earlier identify APD patients who are not optimally controlled with oral treatments. This is an important issue because it has been recently shown that a high percentage of Italian APD patients continue to be treated with their oral standard therapies notwithstanding the use of more than 5 LD doses per day, more than 50% of daily time spent in OFF and a poor QoL in most of them [11].

The OBSERVE-PD (OBSERVational, cross-sEctional PD) multicountry study was designed to assess the proportion of patients with APD in MD clinics in different Countries [12] and the characterization of clinical and non-clinical features based both on physician’s judgment and on Delphi criteria for APD, developed to help clinicians identify patients with APD as well as optimize DAT eligibility [6]. In this study it was shown that 51% of the PD patients visited at the MD Clinics were classified in the advanced stage [12].

In our sub-analysis of the OBSERVE study, we analyzed the local subgroup with the aim of providing the scientific community with new insights on the frequency of the advanced stage identified in the Italian PD patients at the enrolling MD clinics, analyzing also the level of concordance between the physician judgment on the APD identification and the Delphi criteria for APD, and consequently the level of DATs usage in the eligible patients.

## Methods

### Patient selection

This sub-group analysis of the multi-country, cross-sectional, observational OBSERVE-PD study, was performed on data collected on 140 patients included by 9 MD centers in Italy (out of 128 centers worldwide) enrolling consecutive patients with a clinical diagnosis of PD who were either attending a routine clinical visit or were inpatients. Centers were selected based on DAT availability (i.e. LCIG, CSAI, or DBS).

### Study assessments

Demographic information (age, sex, race, patient residence, and caregiver support), presence of cognitive impairment, PD-related information (date of PD diagnosis, referral history, and disease stage based on physician’s judgment), PD treatment history, patient qualification/eligibility for advanced, non-oral therapies according to physician´s judgment, current PD treatments, physician’s assessment of response to current treatment based on motor fluctuations control (complete response, partial response, no response or too early to assess the response), and PD comorbidities were collected.

The patient’s eligibility for DAT was expressed as per clinician’s judgment for potential patient candidacy for DAT. The Unified Parkinson’s Disease Rating Scale (UPDRS) in “On” stage (including Part II-activities of daily living (ADL) and Part III), Part IV-items 32, 33, 34, and 39, and UPDRS-Part V (modified H&Y staging), were assessed during the single visit. Non-motor symptoms (NMS) and QoL were assessed using respectively the Non-Motor Symptom Scale (NMSS) [13] and the 8-item Parkinson’s Disease Quality of Life Questionnaire (PDQ-8) [14].

The physician’s judgment on APD identification and on DAT eligibility were expressed according to the clinician experience and were mainly based on the assessment of motor, NMS and patient’s QoL considering the contraindications for any specific DAT [15].

### Delphi criteria for APD

The Delphi criteria for APD, recently established to help clinicians in the identification of patients with APD and in the evaluation of DAT eligibility, were used to identify the advanced PD stage. Patients were first assessed by the investigator using their clinical judgment for APD and then assessed using the 11 Delphi criteria for APD [6].

Moreover, in patients who were not in treatment with DAT, the adequacy of symptoms control under oral therapy was assessed using the recently described MANAGE-PD (Making Informed Decisions to Aid Timely Management of Parkinson’s Disease) tool, where the positivity to at least one of the following items derived from the Delphi criteria consensus panel (taking oral Levodopa—LD ≥ 5 times daily, ≥ 2 h of OFF time/day, presence of unpredictable fluctuations of motor symptoms, presence of troublesome dyskinesia, and limitations in ≥ 1 activities of daily living) was suggestive of inadequate control [16]. Indeed, MANAGE-PD is a simple screening tool aimed to support healthcare professional’s decision making for the timely management of people with PD based on comprehensive evaluation of frequency and severity of the motor and non-motor symptoms and related disability [16]. The tool was developed using the Delphi Panel criteria and built on a consensus of these indicators by a sample of MD specialists.

### Statistical analysis

Statistical analyses were performed using the SAS® package, version 9.2 (SAS Institute, Cary, NC). Data were summarized using descriptive statistics. The primary endpoint was the proportion of APD patients as judged by physician. The correlation between the Delphi criteria for APD and physicians’ assessments of APD was determined using an additional sensitivity analysis (Cohen’s kappa statistic) by excluding ongoing DAT patients to eliminate any potential bias due to the invasive treatment. Two-sided 95% CIs were provided for comparative end points; CIs and p values (two-sample t test) were calculated for differences between APD and non-advanced PD (non-APD) patients; for categorical variables the comparison between APD and non-APD patients was performed by chi-square.

## Results

### Patients’ characteristics and caregiver status

Among the 2615 PD patients described in the first OBSERVE-PD publication [12], 140 were recruited by 9 Italian MD Centers.

Based on physician’s assessments, among the 140 recruited patients, 60 (42.9%) had APD. Table 1 shows the demographic and clinical characteristics of the Italian APD and non-APD population. No differences were found between APD and non-APD patients regarding age and sex, with the totality of patients living at home, while some differences were observed in the necessity of caregiver support which was required by 75% of APD patients and only by 13.8% of non-APD patients, with respectively 60% and 63.6% in both groups represented by partner/spouse. A difference between the APD and non-APD patients was also observed in the occupational status, being the majority of APD patients retired (73.3%) (Table 1).

**Table 1:** Demographic characteristics, social support, PD clinical characteristics, and referral status of the Italian APD and non-APD patients according to physician judgment

Demographic characteristics and social support	APD (*N* = 60)	Non APD (*N* = 80)
Age, mean ± SD (Range)	67.2 ± 9.5 (37–88)	63.0 ± 11.1 (42–90)
Male; N (%)	35 (58.3%)	48 (60%)
Female; N (%)	25 (41.7%)	32 (40%)
Living at home; N (%)	60 (100%)	80 (100%)
Retired from work; N (%)	44 (73.3%)	34 (42.5%)
Required caregiver support; N (%)	45 (75%)	11 (13.8%)
Caregiver Type; N (%)	*N* = 45	*N* = 11
-Partner/Spouse -Family member/friends -Professional assistance	27 (60%) 17 (37.8%) 11 (24.4%)	7 (63.6%) 4 (36.4%) 1 (9.1%)
PD characteristics		
-Time since PD diagnosis (years); mean ± SD; (Range) -Presence of motor fluctuations; N (%) -Duration of motor fluctuations (years); mean ± SD; (Range)	13.2 ± 6.1 (3–33.2) 56 (93.3%) 6.2 ± 4.7 (1–20) *N* = 55	4.4 ± 3.6 (0.3) 9 (11.3%) 1.1 ± 0.6 (0.2–2) *N* = 9
Severity of Cognitive dysfunction; N (%)		
Mild Moderate Severe Dementia	25 (75.8%) 7 (21.2%) 1 (3%)	13 (81.3%) 3 (18.8%) 0
Referral to MD Center; N (%)	49 (82%)	53 (66%)
Referring Clinician; N (%)	*N* = 49	*N* = 53
-General Practitioner -Neurologist -Geriatrician -Other	19 (39%) 21 (43%) 1 (2%) 8 (16%)	19 (36%) 18 (34%) 2 (4%) 14 (26%)
Reason for referral (Possible multiple answer); N (%)	*N* = 49	*N* = 53
-PD progression -To allow access to DAT -Uncontrolled symptoms -For diagnostic purposes -Other	19 (39%) 16 (33%) 0 13 (27%) 3 (6%)	7 (13%) 0 4 (8%) 37 (70%) 5 (9%)
Time since referral (years); mean ± SD (Range)	6.0 ± 5.1 (0–24.9)	3.3 ± 3.7 (0.1–15.4)

*APD* Advanced Parkinson’s Disease, *DAT* Device-Aided Therapies, *MD* Movement Disorder

APD patients had a longer disease duration (13.2 ± 6.1 years) compared to non-APD (4.4 ± 3.6 years), most of them (93.3%) having motor fluctuations since 6.2 ± 4.7 years compared to non-APD patients (1.1 ± 0.6 years) (Table 1). Many APD (*N* = 53, 88%) and non-APD patients (*N* = 60, 75%) were affected by PD associated NMS. As shown in Table 2, the most frequent of these symptoms in the APD group were represented by cognitive dysfunction (55%), depression (35%), fatigue (25%), and sleep disorders (20%).

**Table 2:** Type of PD associated comorbidities

Symptom^a^ N (%)	APD (*N* = 60)	Non-APD (*N* = 80)	P between groups (chi-square)
Depression	21 (35%)	13 (16.2%)	**0.011**
Cognitive dysfunction	33 (55%)	16 (20%)	** < 0.001**
Sleep Disorders	12 (20%)	8 (10%)	0.096
Fatigue	15 (25%)	4 (5%)	**0.001**
Hypertension	18 (30%)	29 (36.2%)	NS
Cardiac abnormality	14 (23.3%)	22 (27.5%)	NS
Diabetes Mellitus	6 (10%)	2 (2.5%)	NS
Polyneuropathy/Neuropathy	4 (6.7%)	4 (5%)	NS
Chronic Kidney Disease or Insufficiency	2 (3.3%)	0	NS
Chronic Liver Disease or Insufficiency	1 (1.7%)	1 (1.2%)	NS
Chronic Pulmonary Disease	2 (3.3%)	1 (1.2%)	NS
Skin Disease	0	1 (1.2%)	NS
Orthostatism	3 (5%)	2 (2.5%)	NS
Chronic Gastrointestinal Disease	3 (5%)	4 (5%)	NS
Any malignancy	2 (3.3%)	1 (1.2%)	NS
Other	16 (26.7%)	17 (21.2%)	NS
No comorbidity	7 (11.7%)	20 (25%)	NS

In bold the significant values are reported; ^a^Multiple entries were possible

### Referral process and treatment disposition

Forty-nine out of 60 APD patients (82%) and 53 out of 80 (66%) non-APD patients were referred to the MD centers by community physicians, mostly represented by neurologists. The reasons for referral in the APD patients were mainly due to PD progression (39%) or to screening for DAT eligibility (33%), while most of the non-APD patients were referred for diagnostic purposes (70% of the cases) (Table 1). The APD patients were referred to the MD center having a mean disease duration of 6 ± 5.1 years, while the mean referral period for non-APD patients was shorter (3.3 ± 3.7 years).

Patients using only oral or transdermal antiparkinsonian medications were mostly present in the non-APD group (99%), even if this percentage in APD patients was still high (67%; *p* < 0.001 vs non-APD). Independently from the eligibility, 18 out of 60 APD patients (30%) and 1 out of 80 non-APD patients (1%) were on DAT (Table 3). Forty-six per cent of APD patients (27 out of 58) was taking from 3 to 5 current PD treatments per day compared to 33% of the non-APD patients (Table 3).

**Table 3:** Current treatments usage in APD and non-APD patients and treatment response

	**APD (*N* = 60)**	**Non-APD (*N* = 80)**
Current PD oral/transdermal treatments (Possible multiple answers)	*N* = 60	*N* = 80
Oral levodopa/carbidopa or benserazide	55 (92%)	60 (75%)
Oral dopamine agonists	35 (58%)	48 (60%)
Apomorphine patch	2 (3%)	0
Subcutaneous apomorphine rescue injection	2 (3%)	0
COMT-inhibitors	22 (37%)	6 (8%)
MAO-B inhibitors	14 (23%)	41 (51%)
Amantadine	6 (10%)	6 (8%)
Missing	2 (3%)	0
Current oral/transdermal treatment for PD	*N* = 60	*N* = 80
Patients on only oral/transdermal therapy	40 (67%) ^a^	79 (99%)
Patients on ongoing DAT	18 (30%)	1 (1%)
Missing	2 (3%)	0
Number of current oral/transdermal treatments	*N* = 58	*N* = 80
1	12 (21%)	21 (26%)
2	19 (33%)	33 (41%)
3	20 (34%)	24 (30%)
4	4 (7%)	2 (3%)
5	3 (5%)	0
Treatment Response on current treatment (oral/transdermal or DAT)	*N* = 60	*N* = 80
Complete response	12 (20%) ^a^	55 (69%)
Partial response	47 (78%) ^a^	24 (30%)
Too early to assess response	1 (2%)	1 (1%)
Status of DAT in the eligible patients	*N* = 44 (73%) eligible to DAT	*N* = 5 (6%) eligible to DAT (1 Missing)
Ongoing DAT	18/44 (41%)	1/5 (20%)
Decided at visit to start DAT	9/44 (20%)	0
No DAT	17/44 (39%)	3/5 (60%)
Type of DAT on 44 eligible APD and on 5 eligible non-APD patients		
DBS	11 (25%)	1 (20%)
LCIG	7 (16%)	0
CSAI	0	0
Reasons APD patients were not using DAT (Possible multiple answers)	*N* = 17	
Age	2 (12%)	
Patient refusal	5 (29%)	
Patient needs more time to decide	10 (59%)	
Cognitive related issues	2 (12%)	
Comorbidities	2 (12%)	

*DAT* Device Aided Therapy, *DBS* Deep Brain Stimulation, *CSAI* Apomorphine SC infusion, *LCIG* Levodopa-carbidopa intestinal gel, *COMT* (catechol-O-methyltransferase), *MAOB* (Monoamine oxidase type B)

^a^P < 0.001 vs non-APD patients (Chi-square test)

The current PD treatments were similarly distributed in both groups of patients except for COMT-inhibitors which were most frequently used by APD (37%) compared to non-APD patients (8%). Oral levodopa/carbidopa was the most used antiparkinsonian treatment in both groups followed by dopamine agonists (Table 3).

The response to current treatment (both oral and DAT) was complete in 55 (68.8%) non-APD patients, while this percentage of success was limited to only 12 APD patients (20%; p < 0.001 vs non-APD). A statistically significant (p < 0.001) higher percentage of partial response (*N* = 47; 78.3%) for APD patients was reported compared to non-APD patients (*N* = 24; 30%). In a smaller percentage of patients in both groups it was too early to assess a response (1.7% in APD and 1.3% in non-APD patients) (Table 3).

The MANAGE-PD tool was applied to 42 APD patients (16 not eligible to DAT, 17 eligible but not yet treated and 9 patients with planned DAT) and to 78 non-APD patients.

According to this tool, 41 out of 42 APD patients (97.6%) resulted not adequately controlled on oral drugs, while this percentage was limited to only 18 out of 78 non-APD patients (23%).

### Eligibility for DAT according to physician’s judgment

Among the 60 APD patients, 73% (*N* = 44) were considered eligible to DAT; 18 of them (41%) had ongoing DAT (DBS or LCIG), the mean (SD) duration of DBS treatment and LCIG therapy being 58.5 (59.5) months and 12.6 (13.0) months, respectively. In 9 cases (20%) the decision to start DAT was taken during the visit. The reasons for not using or planning DAT in the remaining 17 APD eligible patients were mainly represented by the patient’s need to have more time to decide (59%) or by patient’s refusal (29%) (Table 3).

### Motor and non-motor symptoms and Delphi criteria assessment

**Fig. 1:**
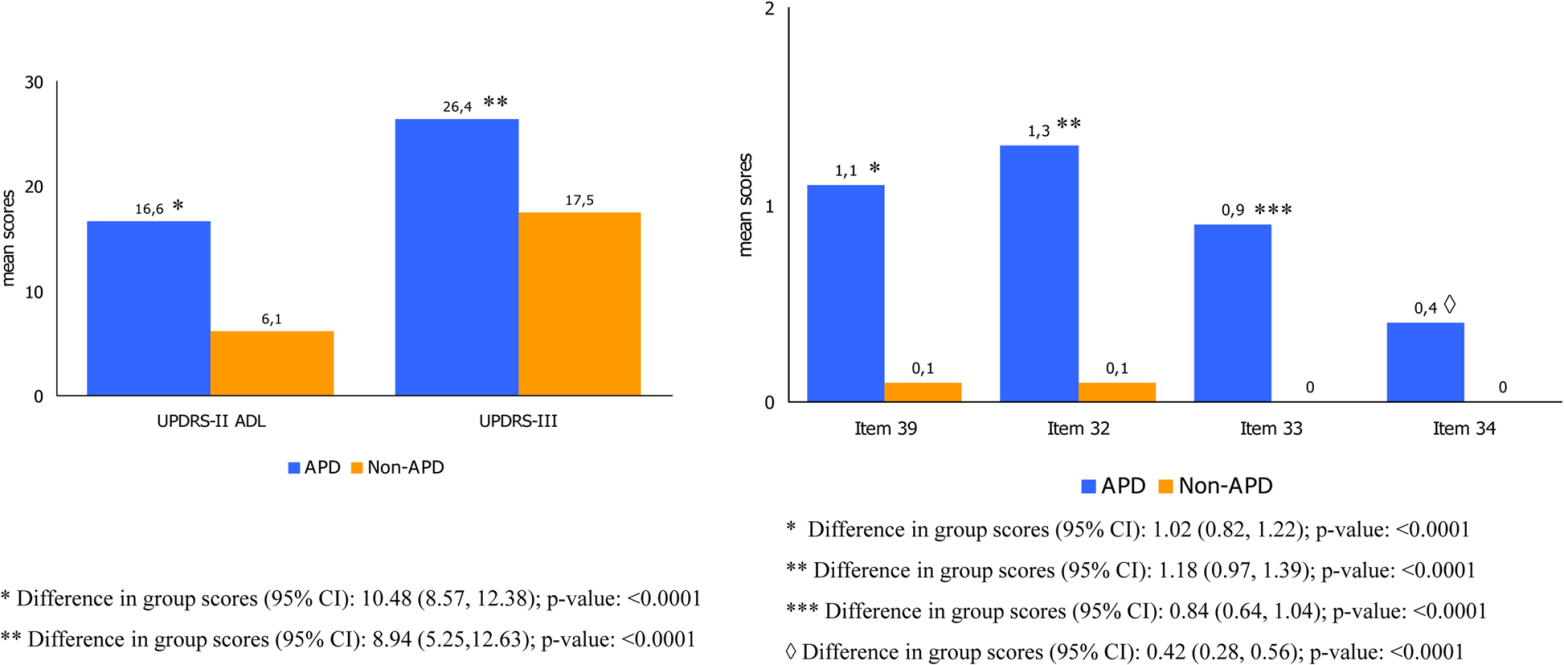
Mean UPDRS-II-ADL, UPDRS-III, and UPDRS-IV sub-items mean scores in APD and non-APD patients. Legend: p values from a paired t test indicate statistical significance; APD = Advanced Parkinsons’ Disease; non-APD = non- Advanced Parkinsons’ Disease; UPDRS-II (Unified Parkinsons’ Disease Rating Scale; ADL (Acitivity of Daily Living); for UPDRS-IV the item 32 to 34 and Item 39 are reported

**Fig. 2:**
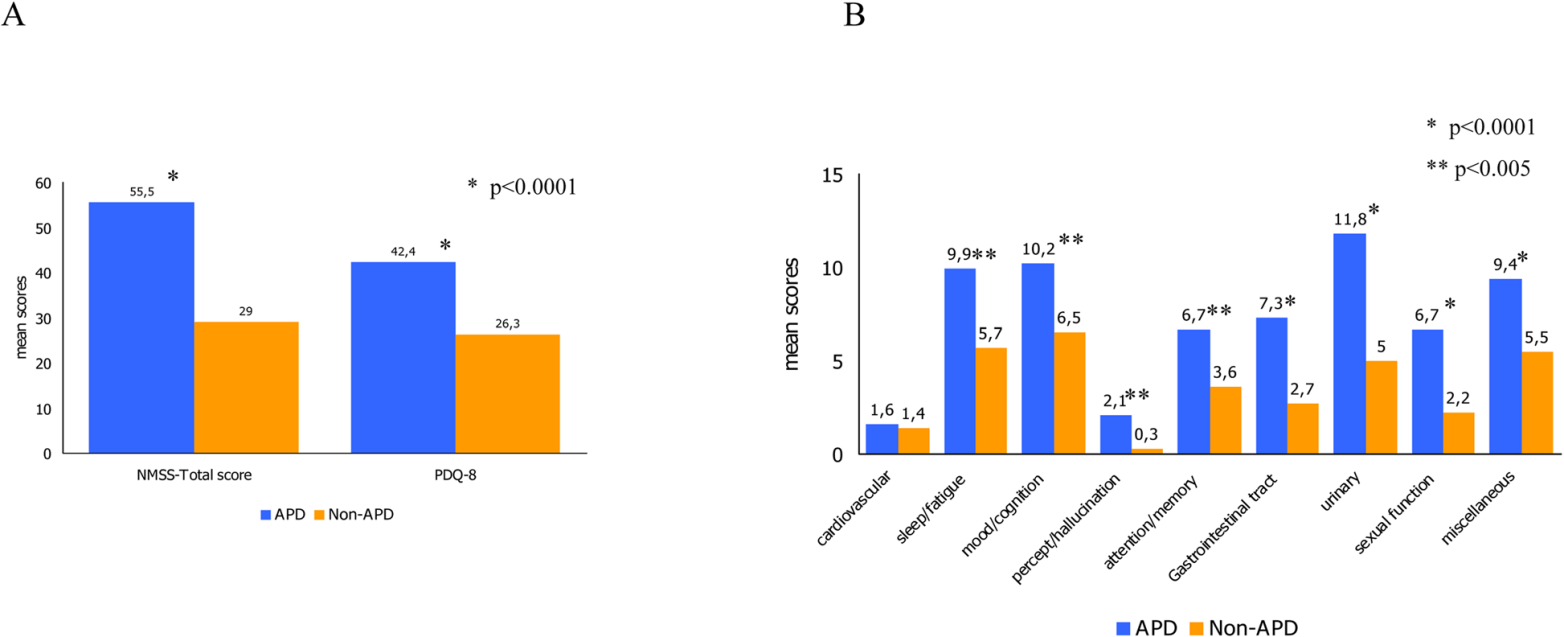
Mean NMSS total (**A**), PDQ-8 (**A**), and NMSS subscores (**B**) in APD and non-APD patients. Figures reported the mean total score of NMSS (**A**), of PDQ-8 (**A**) and the subscore of the NMSS (**B**); p values from a paired t test indicate statistical significance; APD = Advanced Parkinsons’ Disease; non-APD = non- Advanced Parkinsons’ Disease; NMSS = Non-Motor Symptoms Scale; PDQ-8 = Parkinson’s Disease 8-item questionnaire

APD patients showed significantly worse mean scores for UPDRS-part II-ADL, motor symptom severity, dyskinesia duration and disability (items 32, 33 and 34 of the UPDRS-part IV), “Off” time duration (item-39 of the UPDRS-part IV), NMS, and QoL compared to non-APD patients (p < 0.0001 for all), as shown in Fig. 1. Similarly, the NMSS and the PDQ-8 showed higher scores in the APD compared to non-APD patients (Fig. 2).

According to the Delphi criteria, while non-APD patients exhibited no or mild motor fluctuations, 38% of APD patients had moderate to severe motor fluctuations, 30% of them had more than 2 h OFF per day and 45% more than 2 daily hours of dyskinesia (Table 4). Moreover, 56.7% of the APD patients took at least 5 times daily oral levodopa compared to non-APD group (3.8%). Also, the ADL were more limited in the APD group (41.7% moderate or severe) while the majority of the non-APD patients (80%) reported no limitations (Table 4). The overall APD classification made according to physician judgment was consistent to the adjudication according to the Delphi criteria in 93.3% of the cases (Table 5).

**Table 4:** APD Delphi criteria frequency distribution in APD and non-APD patients; N (%)

	**APD (*N* = 60)**	**Non-APD (*N* = 80)**
Severity of Motor Fluctuations		
None	9 (15%)	71 (88.8%)
Mild	28 (46.7%)	9 (11.2%)
* Moderate*	18 (30%)	0
* Severe*	5 (8.3%)	0
Number of OFF hours during waking day		
None	5 (8.3%)	62 (77.5%)
< 2 h	37 (61.7%)	17 (21.3%)
* 2–4 h*	11 (18.3%)	1 (1.2%)
> *4 h*	7 (11.7%)	0
Severity of night sleep disturbances		
None	10 (16.7%)	48 (60%)
Mild	27 (45%)	24 (30%)
* Moderate*	19 (31.7%)	8 (10%)
* Severe*	4 (6.6%)	0
Number of hours with troublesome dyskinesia		
None	14 (23.3%)	68 (85%)
< 2 h	19 (31.7%)	11 (13.8%)
* 2–3 h*	16 (26.7%)	0
> *3 h*	11 (18.3%)	1 (1.2%)
Presence of non-motor fluctuations		
* Yes*	36 (60%)	6 (7.5%)
No	24 (40%)	74 (92.5%)
Presence of OFF time at least every 3 h		
* Yes*	16 (26.7%)	1 (1.3%)
No	44 (73.3%)	79 (98.7%)
Patient took at least 5 times daily oral levodopa		
* Yes*	34 (56.7%)	3 (3.8%)
No	26 (43.3%)	77 (96.2%)
Level of ADL limitation capacity		
None	9 (15%)	64 (80%)
Mild	26 (43.3%)	14 (17.5%)
* Moderate*	18 (30%)	2 (2.5%)
* Severe*	7 (11.7%)	0
Frequency of Falls		
None of the time	21 (35%)	67 (83.7%)
Some of the time	32 (53.3%)	13 (16.3%)
* Most of the time*	6 (10%)	0
* All of the time*	1 (1.7%)	0
Level of dementia		
None	27 (45%)	64 (80%)
Mild	24 (40%)	13 (16.3%)
* Moderate*	8 (13.3%)	3 (3.7%)
* Severe*	1 (1.7%)	0
Level of psychosis		
None	38 (63.3%)	75 (93.8%)
Mild	16 (26.7%)	3 (3.7%)
* Moderate*	5 (8.3%)	2 (2.5%)
* Severe*	1 (1.7%)	0

In italic are reported the level of severity/presence of each symptoms or characteristic that must be present to consider the patient in the advanced stage according to the Delphi Panel criteria approach (5)

**Table 5:** Agreement between physician’s judgment and Delphi criteria for APD (assessed on 60 APD patients)

APD Delphi criteria	Patients with concordance with physician APD classification N (%)	Cohen’s Kappa	95%—CI
Troublesome motor fluctuations, severity level (moderate or severe)	23 (38.3%)	0.415	0.284; 0.546
Off Time, hours/waking day (≥ 2)	18 (30%)	0.314	0.186; 0.443
Night-time sleep disturbances, severity level (moderate or severe)	23 (38.3%)	0.302	0.155;0.448
Troublesome dyskinesia, hours/waking day (≥ 2)	27 (45%)	0.469	0.335;0.603
Presence of non-motor fluctuations	36 (60%)	0.545	0.407; 0.683
Presence of “OFF” time at least every 3 h	16 (26.7%)	0.279	0.154; 0.404
Oral Levodopa dosing ≥ 5 times daily	34 (56.7%)	0.556	0.422; 0.690
Activities of daily living limitation, severity level (moderate or severe)	25 (41.7%)	0.421	0.284; 0.557
Falling frequency (most of the time or all the time)	7 (11.7%)	0.131	0.040; 0.222
Dementia, severity level (moderate or severe)	9 (15%)	0.125	0.015; 0.235
Psychosis, severity level (moderate or severe)	6 (10%)	0.084	-0.009; 0.177
Overall APD classification by Delphi method	56 (93.3%)	0.743	0.633; 0.853

Levels of concordance by Cohen Kappa result interpreted as follows: values ≤ 0 as indicating no agreement; 0.1–0.20 as none to slight; 0.21–0.40 as fair, 0.41– 0.60 as moderate, 0.61–0.80 as substantial; 0.81–1.00 as almost perfect agreement [17]

The correlation between physician’s judgment and the overall APD criteria identified by the Delphi panel was found to be substantial (K = 0.743; 95%CI 0.633–0.853), mainly driven by a moderate concordance found for the Delphi criteria for the presence of ≥ 5 times daily oral LD dosing (K = 0.556; 95%CI 0.422–0.69), the daily time spent with troublesome dyskinesia (K = 0.469; 95%CI 0335–0.603), presence of NMS (K = 0.545; 95%CI 0.407–0.683), and motor fluctuations (K = 0.415; 95%CI 0.284–0.546) (Table 5).

## Discussion

The present study was aimed to describe the prevalence of advanced stage in the Italian OBSERVE-PD population, the patients’ eligibility for DAT, the treatment response as well as the correlation between the clinical judgment of the APD made by physicians with the established Delphi criteria.

The analysis of this Italian cohort showed that among the consecutive PD patients screened, 43% were in the advanced stage and this percentage was even larger in the OBSERVE-PD whole population (51%) [12], and greater than those reported in previous studies [7–9]. This Italian data, as confirmed by the global OBSERVE-PD results, show that APD population could be underestimated and a wider patient population, if early recognized, could benefit from a therapeutic optimization or from device-aided therapies administration that could result in a better control of motor fluctuations and improvement in QoL. Also, Coelho et al. recommended that APD identification should not be substantially related to the H&Y scale, widely used to rank the severity of parkinsonism, while it should be mainly based on the presence of motor complications, the consequent worsening of QoL and reduced independence in ADL and on disease-related or drug-induced NMS [2], differently from previous reports [18]. In the Italian study population, we found a good level of concordance between physician’s judgement and Delphi criteria assessment for APD, where the use of at least 5 times daily oral LD was the criterion with the highest level of concordance. The presence of ≥ 2 h of OFF time and of troublesome dyskinesia were also confirmed to be important clinical determinants of the advanced stage identification, as well as the reduction of ADL and the presence of NMS. This finding is consistent with the recently reported study by Santos-Garcia et al., showing that both health-related and global QoL are worse in patients with 5–2-1 positive criteria, stating that this could be a useful and quick screening assessment to identify APD patients in need of an optimization of PD treatments [19]. Despite the good level of concordance, as also reported in the previous publication on OBSERVE-PD general population [10], our study emphasizes the issue that there still is a gap in current clinical care and possible area for improvement mainly in the timely use of DAT in eligible patients.

Moreover, the screening applying the MANAGE-PD tool revealed that a large amount of APD patients treated with oral therapies were not adequately controlled in their PD symptoms. Our APD patients although having longer disease duration (13.2 years) compared to non-APD patients (4.4 years) and longer duration of motor fluctuations (6.2 vs 1.1 years), were still treated with oral and/or transdermal therapy in 67% of the cases with 78% of them showing a partial response on motor symptoms.

This observation is consistent with the recently published data from PREDICT study reporting that even in the presence of motor fluctuations and disabling dyskinesia, 80% of APD patients continued to be treated with standard oral treatments even if the clinicians reported an unsatisfactory control on motor fluctuations in 88% of the cases and patients were not satisfied of current therapy in 65% of the cases [11].

For this reason, we suggest that the use of an easy tool like the Delphi Panel indicators could help clinicians to identify patients who need treatment optimization or patients who could be eligible for DAT, as recently reported by Santos-Garcia et al. in a Spanish PD population [19]. In the analysis of the global OBSERVE-PD study population the inconsistency in physician judgment for DAT eligibility and the high number of patients who do not receive optimized medical treatment have been underlined [12]. This finding addresses the attention on the importance of an early and correct identification of the advanced stage and of an early assessment of the patient’s eligibility for DATs in order to maintain an acceptable QoL in the long-term.

In our study we found that only 30% of the APD patients were treated with DAT (DBS or LCIG), even if 73% of them were considered eligible for at least one of these treatments. In our Italian sample, CSAI was not used while in the multi-country OBSERVE-PD population it was represented by 8% of the total eligible patients reflecting the less frequent use of this therapeutic approach in Italy [18]. It is interesting to note that in our study the main reason why APD patients did not use a DAT was represented in 88% of the cases by patient’ refusal or by the necessity to have more time to decide. This observation is in line with recently published trials showing that the main reasons driving patients to refuse to be switched from oral drugs to a DAT was the fear of the advanced treatments (56% of the cases) [20] or an excessive anxiety state or poor motivation in 56% [21]. Moreover, the results from a Swedish survey showed that only a small proportion of PD patients were informed about the advanced therapies options by their treating physician and only little more than one in four APD patients had received this information by their doctor [22]. This finding suggests the importance to have a good doctor-patient communication process starting from the diagnosis and continuing along the disease progression, in order to propose to patient the most suitable treatment and guarantee the adherence to therapy [23]. In this view, patient-centeredness is increasingly recognized as a crucial element of quality of care [24, 25]. Patient-centered consultation styles have been associated with higher patients’ satisfaction and improved health outcomes [26]. In fact, dissatisfaction with communication related significantly to non-compliance and it has been reported that PD patients who perceived greater involvement in their care were more satisfied with the consultation and tended to be more compliant [23]. Due to the progressive nature of the disease, patients generally show inadequate adherence to treatment schedules and anti-PD drugs require frequent dose-adjustments and schedule changes, to achieve the best symptomatic control [27]. Malek et al. stated that drug regimens that are simpler and that have fewer daily dosages offer the prospect of better therapy adherence mainly in the later stages of PD when symptom control requires prescription of more than one drug with several daily doses [28, 29]. Moreover, it has been reported that over half of patients treated with at least two anti-parkinsonian medications often take their medications 3–4 times daily, especially APD patients reaching up to 6–10 doses per day [30]. Our investigation confirms this evidence with 46% of the APD patients taking more than 2 oral/transdermal anti-parkinsonian medications compared to 33% of the non-APD patients.

It is also important to consider that the use of DAT can improve adherence due to the reduction of oral therapy coadministration, as shown in the recently published post-hoc analysis of the GLORIA study, where patients using LCIG remained stable in monotherapy for 24 months without any further drug, thus reducing pill burden and potentially leading to greater compliance in APD patients [30].

This evidence is also important considering that most of the APD patients had comorbidities which increased the probability of additional specific treatment needs.

In our study it is interesting to note that, as expected, there was a high frequency of comorbidities such as cognitive dysfunction or depression in APD patients as well as in non-APD. In APD patients these comorbidities could have had an impact on the worse QoL assessed by PDQ-8, UPDRS-part II, and NMSS especially represented by mood/cognition and fatigue. In the previously reported paper by Santos-Garcia QoL has been shown to be worse in patients having Delphi 5–2-1 positive criteria and associated with increased NMS burden and reduced independence in ADL [19]. This issue should be taken into consideration for timely identification of the patients’ progression to APD and the suitability for DAT or therapeutic adaptation as well as the timely referral to MD specialists to improve the quality of care and patient outcomes.

It is also interesting to note that among the comorbidities, both in APD and non-APD patients and independently from the current treatment (DAT or oral/transdermal), the percentage of patients showing polyneuropathy was similar in the two groups (respectively 6.7% and 5%) and this percentage is consistent with that reported by Ceravolo et al. for patients with short LD exposure (6.8%) or non-LD exposure (4.8%) [30].

### Limitations and strengths

This Italian analysis of the OBSERVE-PD study represents a real-world evidence on the characteristics of advanced stage of PD in Italy and it supplies information on the therapeutic approach based on eligibility criteria. This study also provides information on the concordance between the clinical judgment of APD and the Delphi criteria application even if the troublesome dyskinesia criterion was later redefined by the Delphi Panel as ≥ 1 h per day, this representing one of the main limitations [6]. Moreover, the Italian population is part of the total cohort of the original multi-country study and therefore no direct comparison could be performed. For this reason, these results should be taken into account as local descriptive data.

Another limitation of this study is the cross-sectional design that does not allow to catch any information on the possible change of treatment approach based on the application of the Delphi criteria identification and eligibility. Moreover, this study did not collect information on the criteria taken into account by physicians to consider a patient eligible or not to DATs. This study has been conducted in MD centers where the DAT are proposed and implemented, therefore with missing information on general neurologists' attitude towards the screening of advanced stage and referral for DATs. Also, the percentage of APD patients could be overestimated due to the higher number routinely managed in MD Centers rather than in territorial centers. Due to the cross-sectional design, another limitation is represented by the lack of information on the effects on motor and NMS in patients already in treatment with DAT or remaining on oral drugs.

## Conclusions

The analysis of the Italian population of the OBSERVE-PD study confirms that the advanced stage of PD is highly frequent and that most APD patients, even if not adequately controlled by oral therapy and despite their eligibility to DAT, continue to use oral drugs mainly because of fear of DAT or due to patient indecision. Moreover, the high percentage of patients resulting uncontrolled not only by physician judgment but also using the MANAGE-PD screening tool suggests the importance of using a simple and user-friendly tool in clinical practice.

This study has also shown that there is a substantial concordance between the physician judgment on advanced stage identification and the APD identification according to the Delphi criteria, underlying some key criteria for identification, like the daily oral LD doses, the daily hours of OFF time and of troublesome dyskinesia. The possibility to improve the APD patient’s identification may be particularly relevant for optimizing treatment schedules including the consideration for transitioning to device-aided treatment to improve symptoms control and patient’s QoL.

## Data Availability

AbbVie is committed to responsible data sharing regarding the clinical trials we sponsor. This includes access to anonymised, individual and trial-level data (analysis data sets), as well as other information (e.g., protocols and Clinical Study Reports), as long as the trials are not part of an ongoing or planned regulatory submission. This includes requests for clinical trial data for unlicensed products and indications. This clinical trial data can be requested by any qualified researchers who engage in rigorous, independent scientific research, and will be provided following review and approval of a research proposal and Statistical Analysis Plan (SAP) and execution of a Data Sharing Agreement (DSA). Data requests can be submitted at any time and the data will be accessible for 12 months, with possible extensions considered. For more information on the process, or to submit a request, visit the following link: https://www.abbvie.com/our-science/clinical-trials/clinical-trials-data-and-information-sharing/data-and-information-sharing-with-qualified-researchers.html.

## Abbreviations

ADL: Activities of daily living
APD: Advanced Parkinson’s disease
CSAI: Continuous subcutaneous apomorphine infusion
DAT: Device-aided therapies
DBS: Deep brain stimulation
H&Y: Hoehn & Yahr
LCIG: Levodopa-carbidopa intestinal gel
MD: Movement Disorders
NMS: Non-motor symptoms
NMSS: Non-motor Symptom Scale
PD: Parkinson’s disease
PDQ-8: 8-Item Parkinson’s Disease Quality of Life Questionnaire
QoL: Quality of life
UPDRS: Unified Parkinson’s Disease Rating Scale
